# 4-{[Bis(2-hy­droxy­eth­yl)amino]­meth­yl}-6-meth­oxy-2*H*-chromen-2-one

**DOI:** 10.1107/S1600536812030759

**Published:** 2012-07-10

**Authors:** Reshma Naik, Ravish Sankolli, G. N. Anil Kumar, T. N. Guru Row, Manohar V. Kulkarni

**Affiliations:** a P.G. Department of Chemistry, Karnatak University, Dharwad 580 003, India; b Solid State and Structural Chemistry Unit, Indian Institute of Science, Bangalore 560 012, India; c Department of Physics, M.S. Ramaiah Institute of Technology, Bangalore 560054, India

## Abstract

In the title compound, C_15_H_19_NO_5_, an intra­molecular O—H⋯O hydrogen bond links the hy­droxy­ethyl side chains, forming a seven-membered ring. In the crystal, mol­ecules are linked into chains *via* O—H⋯O hydrogen bonds along the *b* axis. Further, mol­ecules are linked by weak inter­molecular C—H⋯O and π–π stacking inter­actions [centroid–centroid distance = 3.707 (4) Å].

## Related literature

For the properties of coumarins, see: Meng *et al.* (1989 ▶); Baures *et al.* (2002 ▶); Jadhav *et al.* (2010 ▶); Basanagouda *et al.* (2011 ▶); Kokila *et al.* (1995 ▶); Khan *et al.* (2008 ▶). For 4-bromo­meth­yl-6-meth­oxy-2*H*-chromen-2-one, see: Basanagouda *et al.* (2011 ▶). For aromatic compounds containing a β-hy­droxy­ethyl side chain, see: Khan *et al.* (2008 ▶). For hydrogen-bond motifs, see: Bernstein *et al.* (1995 ▶). For C—H ⋯O inter­actions, see: Desiraju (2005 ▶). For stacking inter­actions, see: Janiak (2000 ▶). For related literature on 4-bromomethyl-2*H*-chromen-2-one, see: Basanagouda & Kulkarni (2011 ▶).
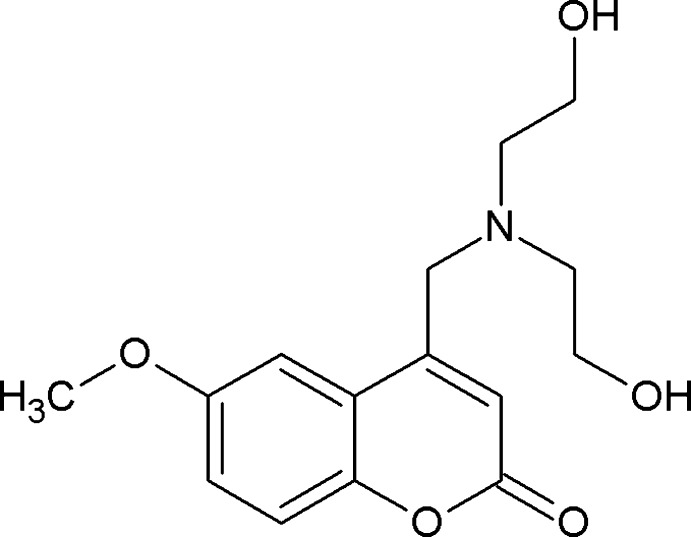


## Experimental

### 

#### Crystal data

C_15_H_19_NO_5_
*M* *_r_* = 293.31
Monoclinic, 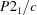

*a* = 9.3038 (5) Å
*b* = 7.9290 (5) Å
*c* = 19.6216 (12) Å
β = 92.944 (5)°
*V* = 1445.56 (15) Å^3^
*Z* = 4
Mo *K*α radiation
μ = 0.10 mm^−1^
*T* = 123 K
0.2 × 0.18 × 0.18 mm

#### Data collection

Bruker SMART APEX CCD detector diffractometer
Absorption correction: multi-scan (*SADABS*; Bruker, 2008 ▶) *T*_min_ = 0.98, *T*_max_ = 0.982
14012 measured reflections
2694 independent reflections
1994 reflections with *I* > 2σ(*I*)
*R*_int_ = 0.040

#### Refinement

*R*[*F* ^2^ > 2σ(*F* ^2^)] = 0.053
*wR*(*F* ^2^) = 0.149
*S* = 1.02
2694 reflections
194 parameters
H-atom parameters constrained
Δρ_max_ = 0.51 e Å^−3^
Δρ_min_ = −0.26 e Å^−3^

### 

Data collection: *SMART* (Bruker, 2008 ▶); cell refinement: *SMART*; data reduction: *SAINT-Plus* (Bruker, 2008 ▶); program(s) used to solve structure: *SHELXS97* (Sheldrick, 2008 ▶); program(s) used to refine structure: *SHELXL97* (Sheldrick, 2008 ▶); molecular graphics: *ORTEP-3* (Farrugia, 1997 ▶) and *CAMERON* (Watkin *et al.*, 1996 ▶); software used to prepare material for publication: *PARST* (Nardelli, 1995 ▶) and *WinGX* (Farrugia, 1999 ▶).

## Supplementary Material

Crystal structure: contains datablock(s) global, I. DOI: 10.1107/S1600536812030759/zj2082sup1.cif

Structure factors: contains datablock(s) I. DOI: 10.1107/S1600536812030759/zj2082Isup2.hkl

Supplementary material file. DOI: 10.1107/S1600536812030759/zj2082Isup3.cml

Additional supplementary materials:  crystallographic information; 3D view; checkCIF report

## Figures and Tables

**Table 1:** Hydrogen-bond geometry (Å, °)

*D*—H⋯*A*	*D*—H	H⋯*A*	*D*⋯*A*	*D*—H⋯*A*
O3—H3*A*⋯O4^i^	0.84	1.85	2.685 (3)	174
O4—H4*A*⋯O3	0.84	2.06	2.875 (3)	164
C7—H7⋯O5^ii^	0.95	2.56	3.494 (2)	167
C14—H14*B*⋯O2^iii^	0.99	2.48	3.206 (3)	130

Symmetry codes: (i) 
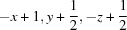
; (ii) 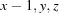; (iii) 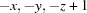.

**Fig. 1.**
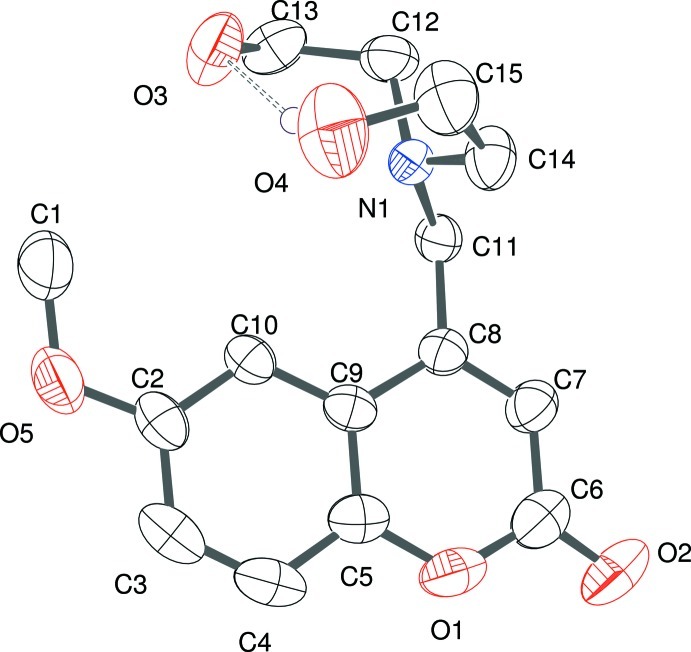
View of the title compound with the atom numbering scheme. Displacement ellipsoids for non-H atoms are drawn at the 50% probability level.

**Fig. 2.**
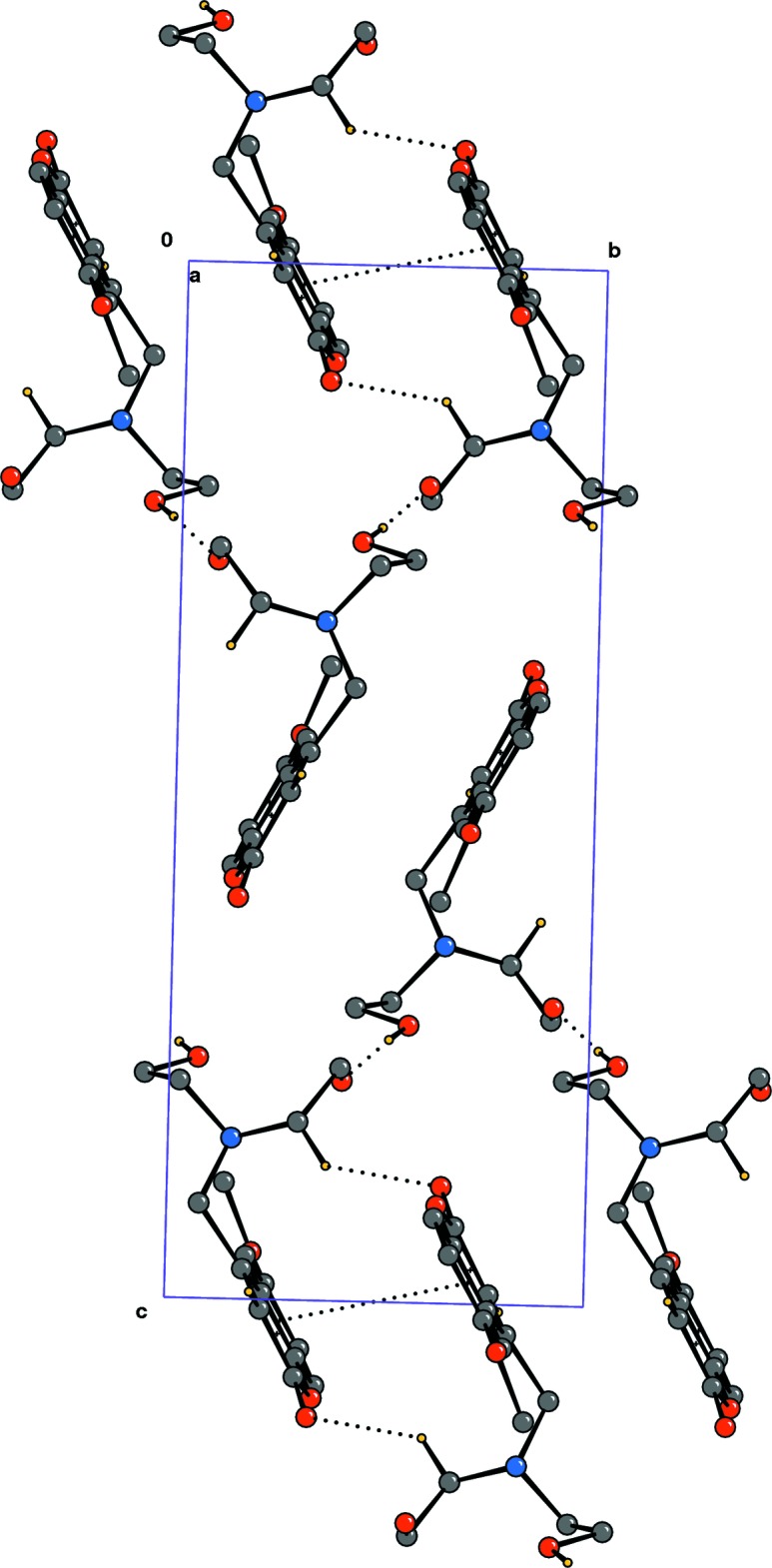
Packing diagram showing intermolecular O—H···O and C—H···O interactions

## supplementary crystallographic information

### Comment

Coumarins have been shown to be important molecular models, revealing different aspects of solid state organic chemistry. Derivatives of coumarin have been investigated for their solid state dimerisation (Meng *et al.*, 1989), self association in the solid state (Baures *et al.*, 2002), and host of other interesting features which have come to light by their crystal structure studies (Jadhav *et al.*, 2010). Our earlier studies have shown that 4-aryloxymethyl coumarins exhibit a Head-tail packing (Gupta *et al.*, 2011), whereas 4-arylamino methyl coumarins exhibit a layer like arrangement (Kokila *et al.*, 1995) in solid state.

Diethanol amines are the key intermediate in the synthesis of nitrogen mustards and there are few reports on the structures possessing β-hydroxy ethyl side chain (Khan *et al.*, 2008). In the light of above observations it was thought of considerable interest to study the title compound (Fig. 1) which was intermediate in a series of Nitrogen mustards synthesised for their anti-cancer activities.

The X-ray crystal structures of the title compound reveal that molecule is non-planar. The diethanol amine side chain is oriented towards the coumarin ring with the nitrogen being out of the plane of the molecule shown by the dihedral angles of C7-C8-C11-N1 -122.53 (2)° and C9-C8-C11-N1 62.23 (2)° . The shortened C2-O5 bond distance of 1.371 (3)Å compared to the C1-O5 bond distance of 1.417 (3) Å indicates the delocalization of lone pair of electrons on the O5 oxygen atom across the coumarin ring. The crystal structure of the compound is stabilized by of both intra and intermolecular O-H···O hydrogen bonds and inter molecular C-H···O interactions. The intramolecular O-H···O hydrogen bond connects diethanol amine side chains. In the solid state, molecules are linked via O-H···O hydrogen bonds. Further molecules are connected by weak C-H···O interactions and π–π stacking interactions [centroid-centroid distance = 3.707 (4)Å symmetry -x,-y,1-z]. (Table1)

### Experimental

The mixture of 4-(bromomethyl)-6-methoxy-2H-chromen-2-one (2.68 g, 0.01 mol) and diethanol amine (1.05 g, 0.015 mol) in a 1:1 (25 ml) mixture of ethylalcohol and ethyl acetate was refluxed for 5-6 h. After completion of the reaction, solvent was removed using rotary evaporator. Obtained viscous mass was diluted with ice cold water and extracted with ethyl acetate and purified by column chromatography using hexane/ ethyl acetate (6:4) as eluting solvent. A slow evaporation technique was used to grow crystals suitable for diffraction studies in ethyl acetate/ hexane (1:1.5) solvent mixture. Light yellow solid; yield 70%; m.p.98-100^o^C; IR (KBr, ν in cm^-1^) 1716 (C=O), 3319 (OH); ^1^H NMR (CDCl_3_, 300 MHz, TMS): δ ppm 2.15 (s, br, 1H, OH, D_2_O exchangeable), 2.82 (t, 4H, J = 6Hz, 2N-CH_2_), 3.34 (s, br, 1H, OH, D_2_O exchangeable), 3.69 (t, 4H, J = 6Hz, 2CH_2_-OH), 3.86 (s, 3H, C_6_-OCH_3_), 3.89 (s, 2H, C_4_-CH_2_), 6.67 (s, 1H, C_3_-H), 7.09 (d, 1H, J = 9Hz, Ar-H), 7.23-7.28 (m, 2H, Ar-H). MS m/z (M+1) 294. Anal. Calcd for C_15_H_19_NO_5 _(%):Calcd. C, 61.42; H, 6.53; N, 4.78. Found: C, 61.28; H, 6.39; N, 4.62.

### Refinement

All H atoms were fixed geometrically and treated as riding with C-H = 0.95Å (aromatic), 0.98Å (methyl) or 0.99Å (methylene) with U_iso_(H) = 1.2U_eq_(C) or U_iso_(H) = 1.5U_eq_(C_methyl_).

### Crystal data

**Table:**

C_15_H_19_NO_5_	*F*(000) = 624
*M**_r_* = 293.31	*D*_x_ = 1.348 Mg m^−^^3^
Monoclinic, *P*2_1_/*c*	Mo *K*α radiation, λ = 0.71073 Å
Hall symbol: -P 2ybc	Cell parameters from 2837 reflections
*a* = 9.3038 (5) Å	θ = 2.8–29.6°
*b* = 7.9290 (5) Å	µ = 0.10 mm^−^^1^
*c* = 19.6216 (12) Å	*T* = 123 K
β = 92.944 (5)°	Block, white
*V* = 1445.56 (15) Å^3^	0.2 × 0.18 × 0.18 mm
*Z* = 4	

### Data collection

**Table:**

Bruker SMART APEX CCD detector diffractometer	1994 reflections with *I* > 2σ(*I*)
Graphite monochromator	*R*_int_ = 0.040
ω scans	θ_max_ = 25.5°, θ_min_ = 2.8°
Absorption correction: multi-scan (*SADABS*; Bruker, 2008)	*h* = −11→11
*T*_min_ = 0.98, *T*_max_ = 0.982	*k* = −9→9
14012 measured reflections	*l* = −23→23
2694 independent reflections	

### Refinement

**Table:**

Refinement on *F*^2^	Secondary atom site location: difference Fourier map
Least-squares matrix: full	Hydrogen site location: inferred from neighbouring sites
*R*[*F*^2^ > 2σ(*F*^2^)] = 0.053	H-atom parameters constrained
*wR*(*F*^2^) = 0.149	*w* = 1/[σ^2^(*F*_o_^2^) + (0.0607*P*)^2^ + 0.6914*P*] where *P* = (*F*_o_^2^ + 2*F*_c_^2^)/3
*S* = 1.02	(Δ/σ)_max_ < 0.001
2694 reflections	Δρ_max_ = 0.51 e Å^−^^3^
194 parameters	Δρ_min_ = −0.26 e Å^−^^3^
0 restraints	Extinction correction: *SHELXL97* (Sheldrick, 2008)
Primary atom site location: structure-invariant direct methods	Extinction coefficient: 0.0048 (14)

### Special details

**Table:**

Geometry. All esds (except the esd in the dihedral angle between two l.s. planes) are estimated using the full covariance matrix. The cell esds are taken into account individually in the estimation of esds in distances, angles and torsion angles; correlations between esds in cell parameters are only used when they are defined by crystal symmetry. An approximate (isotropic) treatment of cell esds is used for estimating esds involving l.s. planes.
Refinement. Refinement of F^2^ against ALL reflections. The weighted R-factor wR and goodness of fit S are based on F^2^, conventional R-factors R are based on F, with F set to zero for negative F^2^. The threshold expression of F^2^ > 2sigma(F^2^) is used only for calculating R-factors(gt) etc. and is not relevant to the choice of reflections for refinement. R-factors based on F^2^ are statistically about twice as large as those based on F, and R- factors based on ALL data will be even larger.

### Fractional atomic coordinates and isotropic or equivalent isotropic displacement parameters (Å^2^)

**Table:**

	*x*	*y*	*z*	*U*_iso_*/*U*_eq_	
C1	0.6985 (3)	0.3637 (5)	0.38763 (14)	0.0794 (9)	
H1A	0.6439	0.2862	0.3572	0.119*	
H1B	0.7954	0.3787	0.3713	0.119*	
H1C	0.6494	0.473	0.3881	0.119*	
C2	0.5821 (2)	0.2628 (3)	0.48505 (12)	0.0478 (6)	
C3	0.5966 (3)	0.1769 (3)	0.54703 (13)	0.0576 (7)	
H3	0.6895	0.1449	0.5648	0.069*	
C4	0.4789 (3)	0.1386 (3)	0.58220 (12)	0.0582 (7)	
H4	0.4892	0.0812	0.6247	0.07*	
C5	0.3436 (3)	0.1843 (3)	0.55550 (11)	0.0477 (6)	
C6	0.0911 (3)	0.1865 (3)	0.57398 (13)	0.0587 (6)	
C7	0.0712 (2)	0.2717 (3)	0.50921 (11)	0.0489 (6)	
H7	−0.0241	0.2978	0.4929	0.059*	
C8	0.1804 (2)	0.3160 (3)	0.47075 (10)	0.0384 (5)	
C9	0.3255 (2)	0.2694 (3)	0.49382 (10)	0.0388 (5)	
C10	0.4483 (2)	0.3085 (3)	0.45839 (11)	0.0403 (5)	
H10	0.4391	0.3666	0.416	0.048*	
C11	0.1511 (2)	0.4227 (3)	0.40816 (10)	0.0396 (5)	
H11A	0.0465	0.446	0.4035	0.048*	
H11B	0.2009	0.5322	0.4151	0.048*	
C12	0.1882 (2)	0.4748 (3)	0.28977 (11)	0.0533 (6)	
H12A	0.1148	0.5608	0.2994	0.064*	
H12B	0.1587	0.419	0.2461	0.064*	
C13	0.3309 (3)	0.5588 (3)	0.28329 (13)	0.0631 (7)	
H13A	0.3242	0.6448	0.2467	0.076*	
H13B	0.3614	0.6154	0.3266	0.076*	
C14	0.1223 (2)	0.1916 (3)	0.32783 (12)	0.0512 (6)	
H14A	0.0226	0.2163	0.3107	0.061*	
H14B	0.1171	0.1226	0.3697	0.061*	
C15	0.1966 (3)	0.0928 (4)	0.27525 (14)	0.0663 (7)	
H15A	0.159	−0.024	0.2744	0.08*	
H15B	0.1741	0.1433	0.2298	0.08*	
N1	0.19564 (16)	0.3493 (2)	0.34476 (8)	0.0376 (4)	
O1	0.2286 (2)	0.1440 (2)	0.59428 (8)	0.0618 (5)	
O2	−0.0017 (2)	0.1540 (3)	0.61251 (10)	0.0857 (7)	
O3	0.43185 (19)	0.4325 (3)	0.26743 (10)	0.0751 (6)	
H3A	0.506	0.4782	0.2532	0.113*	
O4	0.34542 (19)	0.0885 (3)	0.28742 (12)	0.0841 (7)	
H4A	0.3776	0.1875	0.2893	0.126*	
O5	0.70828 (16)	0.2960 (2)	0.45453 (9)	0.0662 (5)	

### Atomic displacement parameters (Å^2^)

**Table:**

	*U*^11^	*U*^22^	*U*^33^	*U*^12^	*U*^13^	*U*^23^
C1	0.0469 (14)	0.131 (3)	0.0604 (17)	−0.0038 (16)	0.0068 (12)	−0.0128 (18)
C2	0.0422 (12)	0.0498 (13)	0.0503 (13)	0.0045 (10)	−0.0086 (10)	−0.0120 (11)
C3	0.0588 (15)	0.0521 (14)	0.0592 (15)	0.0117 (12)	−0.0228 (12)	−0.0096 (12)
C4	0.0791 (18)	0.0484 (14)	0.0449 (13)	0.0037 (12)	−0.0171 (12)	0.0041 (11)
C5	0.0628 (14)	0.0406 (12)	0.0396 (12)	−0.0014 (10)	0.0002 (10)	0.0000 (10)
C6	0.0666 (16)	0.0563 (15)	0.0544 (15)	−0.0097 (13)	0.0150 (13)	0.0012 (12)
C7	0.0491 (12)	0.0529 (13)	0.0453 (13)	−0.0036 (10)	0.0101 (10)	−0.0010 (11)
C8	0.0420 (11)	0.0376 (11)	0.0357 (11)	0.0003 (9)	0.0038 (8)	−0.0056 (9)
C9	0.0465 (12)	0.0340 (11)	0.0354 (11)	0.0012 (9)	−0.0014 (9)	−0.0043 (9)
C10	0.0416 (11)	0.0404 (11)	0.0383 (11)	0.0023 (9)	−0.0026 (9)	−0.0053 (9)
C11	0.0349 (10)	0.0443 (12)	0.0400 (11)	0.0048 (9)	0.0040 (8)	−0.0015 (9)
C12	0.0523 (13)	0.0653 (16)	0.0421 (12)	0.0122 (11)	0.0014 (10)	0.0114 (11)
C13	0.0777 (17)	0.0579 (16)	0.0551 (15)	−0.0041 (14)	0.0164 (13)	0.0162 (12)
C14	0.0431 (12)	0.0556 (14)	0.0556 (14)	−0.0050 (10)	0.0102 (10)	−0.0145 (11)
C15	0.0559 (15)	0.0741 (18)	0.0695 (17)	0.0041 (13)	0.0103 (12)	−0.0231 (15)
N1	0.0332 (8)	0.0461 (10)	0.0336 (9)	0.0010 (7)	0.0036 (7)	0.0016 (7)
O1	0.0804 (12)	0.0614 (11)	0.0441 (9)	−0.0060 (9)	0.0082 (8)	0.0127 (8)
O2	0.0929 (14)	0.0930 (16)	0.0747 (13)	−0.0181 (12)	0.0384 (11)	0.0192 (12)
O3	0.0592 (11)	0.0812 (14)	0.0872 (14)	−0.0104 (10)	0.0272 (10)	0.0099 (11)
O4	0.0599 (11)	0.0792 (14)	0.1143 (17)	0.0198 (10)	0.0140 (11)	−0.0294 (13)
O5	0.0376 (9)	0.0929 (14)	0.0672 (12)	0.0067 (9)	−0.0067 (8)	−0.0087 (10)

### Geometric parameters (Å, º)

**Table:**

C1—O5	1.417 (3)	C10—H10	0.95
C1—H1A	0.98	C11—N1	1.453 (2)
C1—H1B	0.98	C11—H11A	0.99
C1—H1C	0.98	C11—H11B	0.99
C2—O5	1.370 (3)	C12—N1	1.467 (3)
C2—C10	1.374 (3)	C12—C13	1.497 (3)
C2—C3	1.394 (3)	C12—H12A	0.99
C3—C4	1.358 (4)	C12—H12B	0.99
C3—H3	0.95	C13—O3	1.419 (3)
C4—C5	1.386 (3)	C13—H13A	0.99
C4—H4	0.95	C13—H13B	0.99
C5—O1	1.382 (3)	C14—N1	1.455 (3)
C5—C9	1.388 (3)	C14—C15	1.493 (3)
C6—O2	1.204 (3)	C14—H14A	0.99
C6—O1	1.363 (3)	C14—H14B	0.99
C6—C7	1.443 (3)	C15—O4	1.393 (3)
C7—C8	1.343 (3)	C15—H15A	0.99
C7—H7	0.95	C15—H15B	0.99
C8—C9	1.450 (3)	O3—H3A	0.84
C8—C11	1.505 (3)	O4—H4A	0.84
C9—C10	1.402 (3)		
			
O5—C1—H1A	109.5	C8—C11—H11A	108.5
O5—C1—H1B	109.5	N1—C11—H11B	108.5
H1A—C1—H1B	109.5	C8—C11—H11B	108.5
O5—C1—H1C	109.5	H11A—C11—H11B	107.5
H1A—C1—H1C	109.5	N1—C12—C13	110.84 (18)
H1B—C1—H1C	109.5	N1—C12—H12A	109.5
O5—C2—C10	124.2 (2)	C13—C12—H12A	109.5
O5—C2—C3	115.36 (19)	N1—C12—H12B	109.5
C10—C2—C3	120.4 (2)	C13—C12—H12B	109.5
C4—C3—C2	120.5 (2)	H12A—C12—H12B	108.1
C4—C3—H3	119.7	O3—C13—C12	107.7 (2)
C2—C3—H3	119.7	O3—C13—H13A	110.2
C3—C4—C5	119.3 (2)	C12—C13—H13A	110.2
C3—C4—H4	120.3	O3—C13—H13B	110.2
C5—C4—H4	120.3	C12—C13—H13B	110.2
O1—C5—C4	116.4 (2)	H13A—C13—H13B	108.5
O1—C5—C9	122.0 (2)	N1—C14—C15	112.37 (19)
C4—C5—C9	121.6 (2)	N1—C14—H14A	109.1
O2—C6—O1	117.1 (2)	C15—C14—H14A	109.1
O2—C6—C7	126.1 (3)	N1—C14—H14B	109.1
O1—C6—C7	116.7 (2)	C15—C14—H14B	109.1
C8—C7—C6	123.4 (2)	H14A—C14—H14B	107.9
C8—C7—H7	118.3	O4—C15—C14	112.7 (2)
C6—C7—H7	118.3	O4—C15—H15A	109.1
C7—C8—C9	118.5 (2)	C14—C15—H15A	109.1
C7—C8—C11	119.67 (19)	O4—C15—H15B	109.1
C9—C8—C11	121.66 (17)	C14—C15—H15B	109.1
C5—C9—C10	118.31 (19)	H15A—C15—H15B	107.8
C5—C9—C8	117.74 (19)	C11—N1—C14	112.88 (16)
C10—C9—C8	123.94 (19)	C11—N1—C12	110.68 (17)
C2—C10—C9	119.9 (2)	C14—N1—C12	114.28 (18)
C2—C10—H10	120.1	C6—O1—C5	121.58 (18)
C9—C10—H10	120.1	C13—O3—H3A	109.5
N1—C11—C8	115.19 (17)	C15—O4—H4A	109.5
N1—C11—H11A	108.5	C2—O5—C1	117.51 (17)
			
O5—C2—C3—C4	179.4 (2)	C5—C9—C10—C2	0.0 (3)
C10—C2—C3—C4	−0.6 (4)	C8—C9—C10—C2	178.38 (19)
C2—C3—C4—C5	0.7 (4)	C7—C8—C11—N1	−122.5 (2)
C3—C4—C5—O1	−179.1 (2)	C9—C8—C11—N1	62.1 (3)
C3—C4—C5—C9	−0.5 (4)	N1—C12—C13—O3	60.9 (3)
O2—C6—C7—C8	174.7 (3)	N1—C14—C15—O4	43.9 (3)
O1—C6—C7—C8	−3.0 (4)	C8—C11—N1—C14	61.4 (2)
C6—C7—C8—C9	2.9 (3)	C8—C11—N1—C12	−169.10 (17)
C6—C7—C8—C11	−172.6 (2)	C15—C14—N1—C11	−163.4 (2)
O1—C5—C9—C10	178.66 (19)	C15—C14—N1—C12	68.9 (3)
C4—C5—C9—C10	0.1 (3)	C13—C12—N1—C11	94.1 (2)
O1—C5—C9—C8	0.1 (3)	C13—C12—N1—C14	−137.1 (2)
C4—C5—C9—C8	−178.4 (2)	O2—C6—O1—C5	−176.4 (2)
C7—C8—C9—C5	−1.4 (3)	C7—C6—O1—C5	1.6 (3)
C11—C8—C9—C5	174.02 (19)	C4—C5—O1—C6	178.3 (2)
C7—C8—C9—C10	−179.8 (2)	C9—C5—O1—C6	−0.3 (3)
C11—C8—C9—C10	−4.4 (3)	C10—C2—O5—C1	−7.8 (3)
O5—C2—C10—C9	−179.68 (19)	C3—C2—O5—C1	172.2 (2)
C3—C2—C10—C9	0.3 (3)		

### Hydrogen-bond geometry (Å, º)

**Table:**

*D*—H···*A*	*D*—H	H···*A*	*D*···*A*	*D*—H···*A*
O3—H3*A*···O4^i^	0.84	1.85	2.685 (3)	174
O4—H4*A*···O3	0.84	2.06	2.875 (3)	164
C7—H7···O5^ii^	0.95	2.56	3.494 (2)	167
C14—H14*B*···O2^iii^	0.99	2.48	3.206 (3)	130

Symmetry codes: (i) −*x*+1, *y*+1/2, −*z*+1/2; (ii) *x*−1, *y*, *z*; (iii) −*x*, −*y*, −*z*+1.
